# Three Community-Tested Strategies to Increase Hard-to-Reach Older Adults’ Digital Health Literacy

**DOI:** 10.1093/geroni/igaf122.1331

**Published:** 2025-12-31

**Authors:** Paul Freddolino, Fei Sun, Ha-Neul Kim, Ayaka Lingard, AnnMarie Schneider

**Affiliations:** Michigan State University, East Lansing, Michigan, United States; Michigan State University, East Lansing, Michigan, United States; Michigan State University, East Lansing, Michigan, United States; Michigan State University, East Lansing, Michigan, United States; Michigan State University, East Lansing, Michigan, United States

## Abstract

In most parts of the world, technology use among older adults is increasing, yet remains low. Lowest user rates are among the oldest old, less affluent, and less educated groups of older adults. Many older adults suffer from social isolation and loneliness, often alongside multiple chronic diseases. Enhancing digital health literacy, which includes improving digital skills and awareness of the resources available through telehealth, presents a promising strategy to address these issues. This session will highlight the results of a four-year effort to develop and test models designed to increase hard-to-reach older adults’ digital health literacy. Three successful strategies have emerged: 1) Trained volunteer coaches go directly to home delivered meals recipients’ homes for coaching; 2) Coaches present at congregate meal sites and senior center special programs; and 3) Local groups develop unique strategies that fit their preferences and resources, using manuals and videos freely shared with agencies. Process evaluation points to the role of trust in getting technology resistant older adults to be willing to learn about devices and telehealth. Preliminary outcome data from trials using all three models show improved digital literacy and increased telehealth awareness. However, results on loneliness and isolation measures have not shown statistically significant improvement, likely due to relatively positive scores on these measures at baseline and the relatively low intensity of the interventions. Long-term effects may be more likely to appear. Resources developed for the models to support digital literacy among the aging population will be illustrated.

